# Sentinel surveillance of influenza in Burkina Faso: identification of circulating strains during 2010–2012

**DOI:** 10.1111/irv.12259

**Published:** 2014-06-13

**Authors:** Zékiba Tarnagda, Issaka Yougbaré, Abdoul K Ilboudo, Thérèse Kagoné, Armel M Sanou, Assana Cissé, Isaïe Médah, Denis Yelbéogo, Ndahwouh Talla Nzussouo

**Affiliations:** a Institut de Recherche en Sciences de la Santé, Centre National de Référence pour la GrippeBobo-Dioulasso, Burkina Faso; b West African Master Field Epidemiology and Laboratory Training Program (WA FELTP), University of OuagadougouOuagadougou, Burkina Faso; c St. Michael's Hospital LKSKI - Keenan Research CentreToronto, ON, Canada; d Centre MurazBobo-Dioulasso, Burkina Faso; e Direction de la lutte contre la maladie (DLM), Ministère de la santéOuagadougou, Burkina Faso; f Influenza Division, U.S. Centers for Disease Control and Prevention (CDC)Atlanta, GA, USA

**Keywords:** Burkina Faso, influenza-like illness, sentinel surveillance

## Abstract

**Background:**

Although influenza surveillance has recently been improved in some sub-Saharan African countries, no information is yet available from Burkina Faso.

**Objectives:**

Our study was the first to determine the prevalence of influenza viruses circulating in Burkina Faso through a sentinel surveillance system.

**Methods:**

We conducted sentinel surveillance with oropharyngeal (OP) swabs collected from outpatients (1 month to 83 years) from six sites in Bobo-Dioulasso and Ouagadougou, among patients meeting the WHO/CDC case definition for influenza-like illness (ILI; fever ≥38°C, and cough and/or sore throat in the absence of other diagnosis) from July 2010 to May 2012. Influenza viruses were detected by real-time RT-PCR using CDC primers, probes, and protocols.

**Results:**

The first three ILI cases were enrolled each day; of 881 outpatients with ILI enrolled and sampled, 58 (6·6%) tested positive for influenza viruses (29 influenza A and 29 influenza B). Among the influenza A viruses, 55·2% (16/29) were influenza A (H1N1)pdm09 and 44·8% (13/29) were seasonal A (H3N2). No cases of seasonal A/H1N1 were detected. Patients within 0–5 years and 6–14 years were the most affected, comprising 41·4% and 22·4% laboratory-confirmed influenza cases, respectively. Influenza infections occurred during both the dry, dusty Harmattan months from November to March and the rainy season from June to October with peaks in January and August.

**Conclusions:**

This surveillance was the first confirming the circulation of influenza A (H1N1)pdm09, A/H3N2, and influenza B viruses in humans in Burkina Faso.

## Background

The 2009 influenza pandemic was due to novel influenza virus A (H1N1)pdm09 resulting from triple reassortment of avian, swine, and human influenza viruses.^1^ Most strains of influenza virus did not disproportionately infect adults older than 60 years compared with younger people.^2^,^3^ Although limited data exist about influenza-like-illness fatality cases from Africa, a disproportionate number of estimated pandemic deaths may have occurred in this region.^4^,^5^ The burden of influenza in African countries has been traditionally underestimated by health officials. Influenza-like illness affected all parts of Africa,^6^,^7^ which was already struggling with many communicable diseases including malaria, tuberculosis, and HIV. Influenza viruses have been reported in humans and animals in several African countries,^8^–^10^ including H5N1 in domestic poultry and wild birds in Burkina Faso.^9^,^11^ The data on burden and impact of influenza on human health in subtropical regions are limited. Efforts in prevention of influenza need to effectively target these regions before future pandemics.^12^

Influenza outbreaks are annually recurring events with two major subtypes of seasonal influenza A viruses cocirculating in humans (based on Hemagglutinin proteins from H1 and H3) and caused by subtypes A (H1N1)pdm09 and seasonal H3N2.^13^ Influenza activities are predicted and monitored in developed countries. In the Northern Hemisphere, the influenza season can occur between October and May usually peaking in February. Contrarily, in the Southern Hemisphere, the seasonality of influenza is not clearly determined, except in a few countries like South Africa and Australia. In Australia, influenza occurs from May to October and usually peaks in August.^14^ In contrast to low-income settings, in developed countries, influenza is controlled using immunization and disease management.

Preparedness is a key component for successful implementation of disease control and response measures during an influenza pandemic and seasonal outbreak.^15^–^17^ However, developing countries lack sufficient epidemiologic and virologic capacities to support its efforts.^18^,^19^

## Objectives

The aims of our surveillance were to monitor influenza-like illness (ILI), to confirm circulation of influenza viruses, to describe influenza distribution among age-group through our sentinel surveillance platform, and to send specimens to World Health Organization Collaborating Center (WHO CC), CDC, Atlanta, for vaccine strains selection.

## Material and methods

### Surveillance sites and enrollment

**Figure 1:**
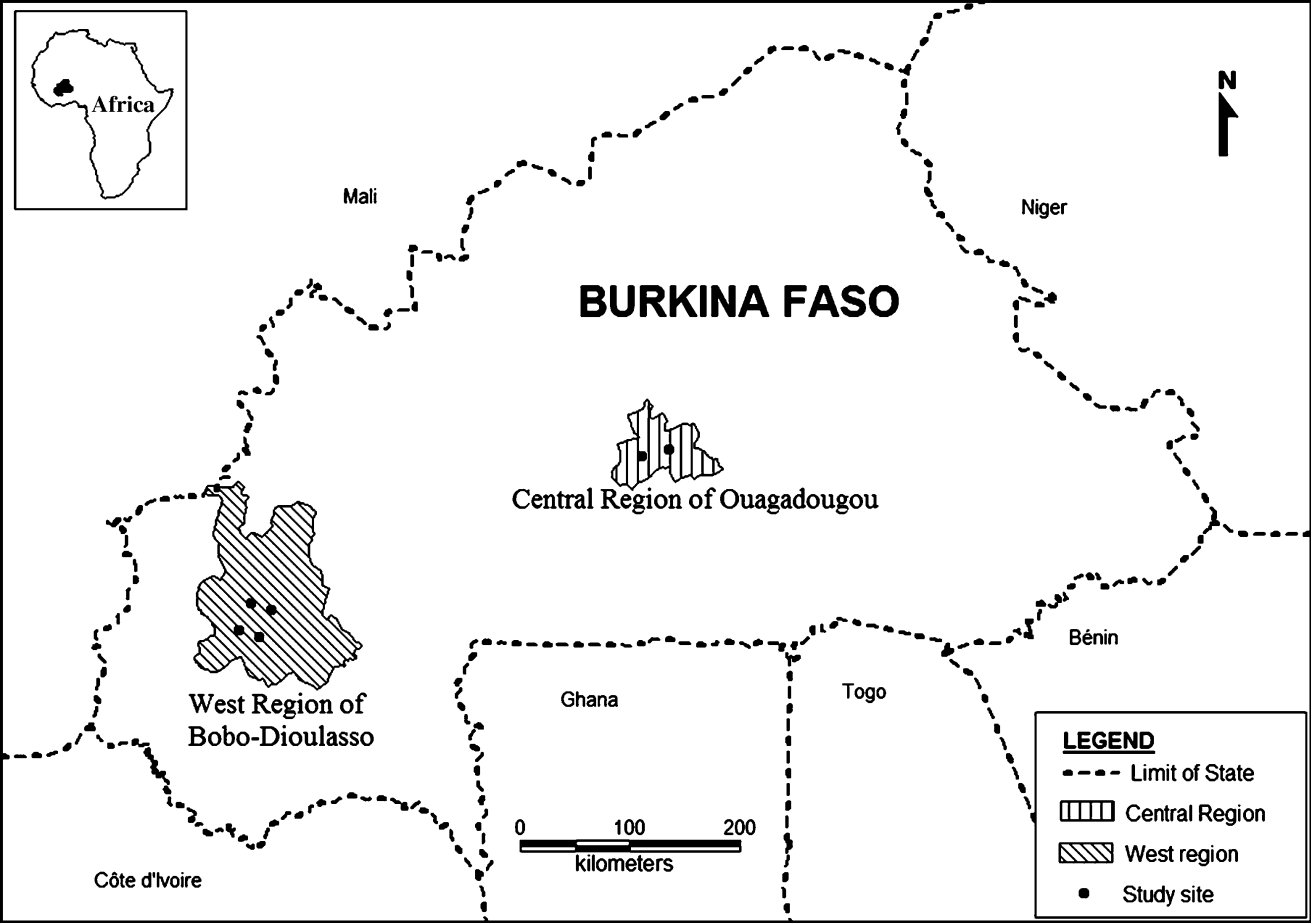
Influenza-like illness surveillance in Burkina Faso: sentinel sites, 2010–2012. Central Region of Ouagadougou: the medical center of the military garrison Guillaume Ouedraogo; the medical center of the military garrison Sangoule Lamizana. Western Region of Bobo-Dioulasso: the district hospital of Do; the medical center of Colsama; the medical center of Bolomakote; the military garrison hospital Ouezzin Coulibaly

As this sentinel surveillance was a public health program implemented by the Burkina Faso Ministry of Health, there was no need of ethical approval. All patients who participated in this surveillance provided informed consent in accordance with Helsinki recommendation. The first 2 years of sentinel surveillance (June 2010–May 2012) involved six sentinel sites (designated by the Ministry of Health) located in the two largest cities of Burkina Faso, the capital city Ouagadougou and Bobo-Dioulasso (Figure1). These sites were selected based on the following criteria: geographic representation within the country; high number of patients consulting at health facilities; the accessibility of the site; the availability and the desire of the physicians or nurses to participate voluntarily in the surveillance program; and availability of a refrigerator (+4°C) for the storage of specimens.

As this surveillance was part of the public health program implemented by the Ministry of Health, health practitioners, physicians, or nurses at the sentinel sites were trained on respiratory symptoms screening, specimen collection, storage, and transportation. Sentinel site staff screened suspected ILI cases based on WHO/CDC and national influenza surveillance ILI case definition (temperature ≥38°C, and cough and/or sore throat in the absence of other diagnosis). Because limited resources were limited to run this sentinel surveillance, only the first three consenting ILI cases of the day, from Monday to Thursday, were enrolled for the surveillance. For each ILI case, standard information was collected using a questionnaire, including the patient's name, sex, age, symptoms, date of onset of illness (3–7 days after onset of fever), vaccination status, and sample collection date. An oropharyngeal specimen was collected from each ILI case using universal transport medium (Copan, Italy) swabs. Samples were stored at +4°C (no longer than 48 hours) and transported twice a week (Tuesday and Thursday) to the influenza national reference laboratory (located in Bobo-Dioulasso) where analyses are performed on Friday and Saturday.

### Samples analysis

In the influenza reference laboratory, each sample was divided into three aliquots in 1·8-ml cryovial tubes. Two aliquots were stored at −80°C immediately. The third aliquot was analyzed by the end of the week by real-time RT-PCR in ABI 7500 Fast thermal cyclers (Applied Biosystems, Foster City, CA, USA) using CDC, Atlanta, primers, probes, and protocol^20^ for influenza A and influenza B detection followed by influenza A subtyping for H1N1pdm09, seasonal H1N1, and seasonal H3N2. Eight positive specimens for influenza viruses (four influenza B viruses, three influenza A (H1N1)pdm09 virus isolates, and one H3N2 virus) were sent to the World Health Organization Collaborating Center (WHO CC), CDC, Atlanta, for vaccine strains selection and other analyses: hemagglutination inhibition tests with a panel of post- infection ferret antisera for detailed antigenic characterization; a functional neuraminidase inhibition assay and/or genetic analysis to assess susceptibility of these viruses to the neuraminidase inhibitors oseltamivir and zanamivir; in addition, sequence analysis (HA, NA, and M genes for type A viruses and HA, NA, and NS genes for type B viruses) was performed.

### Data analysis

Statistical analysis was performed using STATA11.0 (Stata Corporation 12.1, MP. Parallel Edition, College station, TX, USA) and GraphPad Prism was used for mapping. Descriptive analyses comprised assessing frequency distributions and proportions for each variable category. Fisher's exact test for categorical variables was used for group comparisons and anova for continuous variables. Logistic regression analysis was performed to measure the association between ILI or laboratory results and each independent variable (symptom, age-group). Odds ratios (ORs) and 95% confidence intervals (CIs) were calculated. *P* values <0·05 were considered to be statistically significant.

## Results

### Characteristic of population and influenza symptoms

We stratified the 881 outpatients with ILI by age-groups. The participant's median age was 14·3 years [range, 1 month–83 years]. None of the participants to this 2-year surveillance was vaccinated against influenza. Infants from the age-group 1–5 years (29·6%), and those from the age-group 15–34years old (31·2%) were the most numerous (Table1). The majority of the participants (95%) came from suburban areas of the second largest city of Burkina Faso (Bobo-Dioulasso). Except fever and cough, which were present in all ILI cases according to the case definition, sore throat was found in 56% of patients, while vomiting and diarrhea were observed in 19·6% and 7·2%, respectively (Table1). However, after logistic regression analysis, we did not find any relationship between influenza positivity and each of these three symptoms (sore throat: OR=1·27; *P* = 0·47; vomiting: OR=1·30; *P* = 0·46; diarrhea: OR=1·51; *P* = 0·37).

**Table 1:** Influenza-like illness (ILI) symptoms by age-group, Burkina Faso, 2010–2012

Age	ILI cases *n* = 881 (%)	Fever *n* = 881 (%)	Cough *n* = 859 (%)	Sore throat *n* = 494 (%)	Vomiting *n* = 173 (%)	Diarrhea *n* = 64 (%)
<1	137 (15·5)	137 (15·5)	137 (15·9)	17 (3·4)	53 (30·6)	28 (43·8)
1–5	261 (29·6)	261 (29·6)	260 (30·2)	81 (16·4)	72 (41·6)	30 (46·8)
6–14	105 (11·9)	105 (11·9)	99 (11·5)	73 (4·8)	16 (9·2)	1 (1·5)
15–34	275 (31·2)	275 (31·2)	263 (30·6)	241 (48·8)	23 (13·2)	4 (6·2)
35–50	56 (6·4)	56 (6·4)	55 (6·4)	51 (10·3)	8 (4·6)	1 (1·5)
>50	28 (3·2)	28 (3·2)	26 (3·0)	23 (4·6)	1 (0·5)	0 (0·0)
Missing age	19 (2·2)	19 (2·2)	19 (2·2)	8 (1·6)	0 (0·0)	0 (0·0)

### Detection of human influenza viruses in Burkina Faso

Fifty-eight of 881 outpatients with ILI (6·6%, 95% CI: 4·9–8·2) tested by real-time PCR were positive for influenza virus. All age-groups except the group of 35–50 years old had PCR-confirmed cases, with the majority (93%) positives occurring in those <35 years old (Table2). Among PCR positives, patients with fever, cough, and sore throat were present in all age-groups ≥1 year.

**Table 2:** Influenza-like illness (ILI) symptoms of walk-in patients with positive PCR result, by age-group, Burkina Faso, 2010–2012

Range of age (years)	ILI cases *n* (%)	PCR Positive cases *n* (%)	Clinical symptoms of influenza infected patients				

			Fever *n* (%)	Cough *n* (%)	Sore throat *n* (%)	Vomiting *n* (%)	Diarrhea *n* (%)
<1	137 (15·5)	4 (6·9)	4 (6·9)	4 (6·9)	0 (0·0)	2 (16·1)	0 (0·0)
1–5	261 (29·6)	20 (34·5)	20 (34·5)	20 (34·5)	7 (19·4)	6 (50·0)	2 (100·0)
6–14	105 (11·9)	13 (22·4)	13 (22·4)	1 (22·4)	10 (27·8)	3 (25·0)	0 (0·0)
15–34	275 (31·2)	17 (29·3)	17 (29·3)	17 (29·3)	15 (41·7)	1 (8·3)	0 (0·0)
35–50	56 (6·4)	0 (0·0)	*(*)	*(*)	*(*)	*(*)	*(*)
>50	28 (3·2)	3 (5·2)	3 (5·2)	1 (1·7)	3 (8·3)	0 (0·0)	0 (0·0)
Missing age	19 (2·2)	1 (1·7)	1 (1·7)	1 (1·7)	1 (2·8)	0 (0·0)	0 (0·0)
Total	881 (100)	58 (100)	58 (100	58 (100)	36 (62·1)	12 (20·7)	2 (3·5)

* No data are completed concerning the range of 35–50 years because no PCR positive cases of influenza were found in this age range.

Within the 58 positive cases for influenza, 29 cases were influenza A positive and the other 29 cases were influenza B positive (Table3). After subtyping the influenza A viruses, 55·2% (16/29) cases were influenza A (H1N1)pdm09 and 44·8% (13/29) cases were seasonal A/H3N2. No cases of seasonal H1N1 were detected.

**Table 3:** Circulation of influenza A (H1N1)pdm09 and seasonal H3N2) and influenza B virus in Burkina Faso, 2010–2012

Range of age	% of influenza A (H1N1)pdm09 (*n* = 16)	% of influenza A (H3N2) (*n* = 13)	% of influenza B (*n* = 29)
<1	1 (6·2)	1 (7·6)	2 (6·9)
1–5	3 (18·7)	7 (53·8)	9 (31·0)
6–14	5 (31·2)	0 (0·0)	8 (27·5)
15–34	6 (37·5)	3 (23·0)	8 (27·5)
35–50	0 (0·0)	0 (0·0)	0 (0·0)
>50	1 (6·2)	1 (7·6)	1 (3·4)
Missing age	0 (0·0)	1 (7·6)	1 (3·4)

With regard to the eight specimens sent to WHO CC, CDC, Atlanta, results were as follows: From the four influenza B viruses, two viruses belonged to B/Victoria/2/87 lineage and were antigenically similar to the B/Brisbane/60/2008 virus, the two other influenza B viruses were B/Yamagata lineage viruses; the three influenza A (H1N1)pdm09 virus isolates were antigenically characterized as A/California/07/2009-like and their sequences HA fall into genetic group 8 and are very similar to other A (H1N1)pdm09 viruses isolated in 2011 from African region; the H3N2 virus was antigenically similar to A/Victoria/361/2012 virus, which was the H3 component of 2012–2013 influenza vaccine for the Northern Hemisphere and 2013 influenza vaccine for the Southern Hemisphere. All eight detected viruses were sensitive to oseltamivir and zanamivir.

Children of pre-school and school age (1–5 years and 6–14 years) were most affected, respectively, 34·5% and 22·4% of laboratory-confirmed influenza (*P* = 0·01). No positive cases were found among adults of 35–50 years old.

Influenza A (H1N1)pdm09 affected primarily patients of age-groups 6–14 years (31·2%) and 15–34 years (37·5%), while H3N2 infection was most detected among those 1–5 years of age (53·8%) (Table3). Influenza B infection occurred predominantly in three age-groups: 1–5, 6–14, and 15–34 years (*P* = 0·12).

### Pattern of circulation of human influenza viruses in Burkina Faso

**Figure 2:**
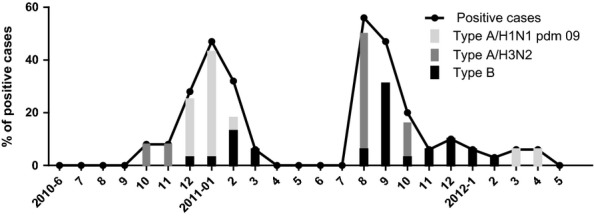
Seasonal trend of influenza-like illness in Burkina Faso, 2010–2012. Three peaks of influenza occurred: the first due to virus influenza A (H1N1)pdm09 from November 2010 to March 2011; the second and the third due to influenza A (H3N2) and influenza B from July 2011 to November 2011. In 2012, smaller numbers of influenza A H1N1pdm 09 and influenza B were detected. NB. In Burkina Faso, the dry, dusty Harmattan season occurs from November to March, and the rainy season, from June to October.

In Burkina Faso, the dry Harmattan season occurs from November to March, and the rainy season, from June to October. The influenza peak during Harmattan (November 2010–March 2011) was predominantly caused by influenza A virus, whereas the second peak during the rainy season of 2011 (which occurred from June to October) was caused by a combination of influenza A and B viruses (Figure2). No influenza peak during the 2012 Harmattan was observed. No case of seasonal H1N1 was found during the 2-year surveillance.

### Study limitations

This is the first influenza surveillance platform set up in Burkina Faso country, where influenza was thought to be less prevalent, therefore somehow neglected, and this was a key reason to mobilize human and material resources to effectively start the surveillance in order to better understand the epidemiology of influenza in Burkina Faso.

The medical staff was trained to collect oropharyngeal or nasopharyngeal swabs samples from patients with ILI and SARI; unfortunately, this surveillance platform relied on oropharyngeal swabs only and not nasopharyngeal swabs, and that might have influenced the prevalence of influenza which was as low as 6·6%.

In the field, we observed that people over 50 years did not like to seek medical care in health facilities for flu.

Our sample size is small, and conducting only 2-year surveillance is not enough to have substantial data to conclude any seasonality or any complete molecular characterization.

## Discussion

The influenza A/H1N1 pandemic occurred in November 2010 through March 2011 in Burkina Faso, because of the delayed introduction of the 2009 influenza pandemic virus into West Africa.^21^ Until recently, influenza infection was largely unknown by common people in Africa, and it continues to be a low priority for Governments and public health officials.^18^,^19^ When influenza A/H5N1 was reported across the continent for the first time in wild and domestic birds,^11^,^22^ the general speculation was that the disease could not be transmitted to humans. In 2009, public health experts were concerned about a severe outbreak of pandemic influenza in Africa because the continent was not well prepared.^19^,^23^,^24^ We conducted 2-year sentinel surveillance to monitor influenza-like illness (ILI), to confirm circulation of influenza viruses, to describe influenza distribution among age-group through our sentinel surveillance platform, and to send specimens to WHO CC, CDC, Atlanta, for vaccine strains selection.

This is the first report of human influenza in Burkina Faso. We found an influenza prevalence of 6·6% among enrolled persons seeking care for ILI. More than 50% of influenza infections were in persons <15 years old. In developed countries, the most common symptoms are fever, cough, sore throat, runny nose, muscle pains, and weakness/fatigue.^16^ Because people with other infectious respiratory diseases can present with the same symptoms during the same season, conducting surveillance for a longer period will help to better understand the seasonality of influenza.

West African Sub-Saharan countries experience Harmattan weather patterns with a dry and dusty wind that occurs between November and March; this weather does have a public health impact, especially on respiratory diseases and meningitis.^25^,^26^ Influenza A and B viruses circulated with equal prevalence. However, during this 2-year surveillance, influenza type A was predominant during the dusty Harmattan and influenza type B during the rainy season. Because influenza vaccine is neither available nor mandatory and antiviral drugs are used very sparsely in Burkina Faso, these data will assist decision-makers in the preparation of adequate response to seasonal influenza outbreaks, pandemics, and vaccine policy development. Priority should be given to immunization of children ≤5 years, because in our data, this age-group represents more than 40% of total influenza positive cases. One additional benefit of the surveillance was to raise clinician awareness about influenza. The implementation of national influenza centers in several West African countries for routine surveillance with the help of the World Health organization (WHO) and the Centers for Disease Control and Prevention (CDC) is a key step for better understanding and control of influenza disease in sub-Saharan Africa.^7^,^10^,^27^

## Conclusion

During 2010–2012, the subtypes A (H1N1)pdm09 and Seasonal H3N2 of influenza A virus and influenza B virus cocirculated in Burkina Faso. Only eight influenza strains from our country were tested for antiviral susceptibility, and all were sensitive to oseltamivir and zanamivir.

The sentinel surveillance data allowed description of the pattern of disease activity. Both seasonal and pandemic influenza surveillance, using sentinel data, are informative when combined with laboratory testing. We advocate enhancing the surveillance of influenza in Burkina Faso to build up and sustain an influenza surveillance system as well as pandemic preparedness capacities.

Future surveillance should combine virologic, epidemiologic, and environmental data to understand factors that drive influenza virus activity and circulation at both national and regional levels.
